# Talking about quality: exploring how ‘quality’ is conceptualized in European hospitals and healthcare systems

**DOI:** 10.1186/1472-6963-14-478

**Published:** 2014-10-11

**Authors:** Siri Wiig, Karina Aase, Christian von Plessen, Susan Burnett, Francisco Nunes, Anne Marie Weggelaar, Boel Anderson-Gare, Johan Calltorp, Naomi Fulop, For QUASER-team

**Affiliations:** Department of Health Studies, University of Stavanger, N-4036 Stavanger, Norway; Department of Pulmonary and Infectious Diseases, North Zealand Hospital, Dyrehavevej, 29-3400 Hilleroed, Denmark; Imperial College, London, St Mary’s Campus, Norfolk Place, London, W2 1PG UK; ISCTE, Lisboa, Instituto Superior de Ciências do Trabalho e da Empresa (ISCTE), Av.ª das Forças Armadas, Lisbon, 1649-026 Portugal; Department of Health Policy and Management, Erasmus University Rotterdam, Postbus 1738, 3000 DR Rotterdam, The Netherlands; Jönköping Academy for improvement of Health and Welfare, Jönköping University, Box 1026, 551 11 Jönköping, Sweden; Department of Applied Health Research, University College London, 1-19 Torrington Place, London, WC1E 7HB UK

**Keywords:** Quality conceptualization, Clinical effectiveness, Patient safety, Patient experience, Multi-level case study, Quality improvement

## Abstract

**Background:**

Conceptualization of quality of care – in terms of what individuals, groups and organizations include in their meaning of quality, is an unexplored research area. It is important to understand how quality is conceptualised as a means to successfully implement improvement efforts and bridge potential disconnect in language about quality between system levels, professions, and clinical services. The aim is therefore to explore and compare conceptualization of quality among national bodies (macro level), senior hospital managers (meso level), and professional groups within clinical micro systems (micro level) in a cross-national study.

**Methods:**

This cross-national multi-level case study combines analysis of national policy documents and regulations at the macro level with semi-structured interviews (383) and non-participant observation (803 hours) of key meetings and shadowing of staff at the meso and micro levels in ten purposively sampled European hospitals (England, the Netherlands, Portugal, Sweden, and Norway). Fieldwork at the meso and micro levels was undertaken over a 12-month period (2011–2012) and different types of micro systems were included (maternity, oncology, orthopaedics, elderly care, intensive care, and geriatrics).

**Results:**

The three quality dimensions clinical effectiveness, patient safety, and patient experience were incorporated in macro level policies in all countries. Senior hospital managers adopted a similar conceptualization, but also included efficiency and costs in their conceptualization of quality. ‘Quality’ in the forms of measuring indicators and performance management were dominant among senior hospital managers (with clinical and non-clinical background). The differential emphasis on the three quality dimensions was strongly linked to professional roles, personal ideas, and beliefs at the micro level. Clinical effectiveness was dominant among physicians (evidence-based approach), while patient experience was dominant among nurses (patient-centered care, enough time to talk with patients). Conceptualization varied between micro systems depending on the type of services provided.

**Conclusion:**

The quality conceptualization differed across system levels (macro-meso-micro), among professional groups (nurses, doctors, managers), and between the studied micro systems in our ten sampled European hospitals. This entails a managerial alignment challenge translating macro level quality definitions into different local contexts.

## Background

Quality improvement (QI) requires collaboration and alignment between professionals, managers (clinical and non-clinical), and policy makers from different disciplines and levels in healthcare systems across Europe [1, 2]. This is challenging because these actors may have different concerns and goals and use different professional vocabularies regarding the quality of care.

Diverse definitions of quality have been developed and often include dimensions such as effectiveness, timeliness, safety, equity, efficiency, and patient centeredness [3, 4]. Conceptualization of the quality of care – in terms of what individuals, groups and organizations include in their meaning of quality, (e.g. the perspectives, defining attributes, quality dimensions, contextual factors, dilemmas) is relatively unexplored in health services research [5–7]. Previous research shows differences in quality conceptualizations between professionals and hospital managers [1, 5, 8], within professional groups [7, 9, 10], between patients and relatives [11], and between patients and doctors [12]. Research from Australia showed differences in professionals’ perspectives on quality and found that administrative staff focused on emotional labour associated with protecting the patient; nurses had a “practitioner” viewpoint highlighting their central role in care provision; doctors’ concerns were directly associated with professional competence and improvement of skills; while hospital managers had the most complex conceptualization of quality incorporating a broader picture including risks, reporting, and improvement strategies [5]. Studies of the macro level pinpoint how national and international institutions, such as OECD, Department of Health (UK) conceptualize quality through measurements and classification frameworks [13]. It has been argued that differences within organizations, between professional groups and hospital managers about how to improve quality may impede the successful implementation of quality programs [1]. However, to date there is still a lack of cross-national studies with a multi-level perspective on how quality is conceptualized in healthcare.

### Aim and research question

This study develops the discussion of the relevance of shared conceptualization in implementation of QI in healthcare. The overall aim is to explore quality conceptualization in a multi-level (macro-meso-micro) perspective and discuss implications for senior hospital managers, as this group is a key in translating knowledge into practice [14, 15]. More precisely, we explore and compare conceptualization of quality among national bodies (macro level), senior hospital managers (meso level), and professional groups in clinical micro systems (micro level) in a cross-national study. This paper addressed the following research question:

*How is ‘quality’ conceptualized at the macro-, meso- and micro-levels in a selection of hospitals in the healthcare systems of five European countries?*

By identifying how the conceptualization of quality varies across the national, hospital, and micro system levels, and between professionals and managers, the study contributes to a better understanding of the requirements for improved QI processes.

## Methods

This study is part of the EU FP7 project *Quality and Safety in European Union Hospitals (QUASER)* [14]. Two central features of QUASER are 1) to study ‘quality’ from a multi-level perspective; and 2) the working definition of quality (showed in Table 1), incorporating three dimensions: Clinical effectiveness (CE), patient safety (PS), and patient experience (PE) [14, 16].

**Table 1:** **QUASER project definition of quality dimensions**

Clinical effectiveness (CE)	Patient safety (PS)	Patient experience (PE)
The degree to which healthcare services for individuals and populations increase the likelihood of desired health outcomes and are consistent with current professional knowledge [17].	The avoidance, prevention and amelioration of adverse outcomes or injuries stemming from the process of health care [18].	Eight dimensions of “patient-centred care” [19]:
		1. Fast access to reliable health advice
		2. Effective treatment delivered by trusted professionals
		3. Clear, comprehensible information and support for self-care
		4. Involvement in decisions and respect for patient preferences
		5. Attention to physical and environmental needs
		6. Emotional support, empathy and respect
		7. Involvement of, and support for, family and carers
		8. Continuity of care and smooth transitions.

The widely adopted definition of quality by the Institute of Medicine (IOM) [3, 4] includes six quality dimensions (effectiveness, timeliness, safety, equity, efficient, and patient centeredness). Our working definition of quality drew on the Darzi definition [20] and the European Commission Framework Programme 7 Health Work Programme 2009 (EU FP7) including CE, PS, PE as the main quality dimensions [21].

### Design and sample

The study is based on multi-level longitudinal comparative case studies in 10 hospitals in five countries (England, Portugal, Sweden, Norway, and the Netherlands) (see QUASER study protocol for detailed information about design, methods, procedures, and analysis [14]). The countries represent different types of European healthcare and funding systems. Hospitals were selected and approached according to an agreed hospital selection framework based on performance on quality indicators (see Burnett et al. [22] for detailed information about the hospital selection framework, criteria, rationale, and process). The purpose of the selection process was to find hospitals at different stages of the quality journey. One hospital in each country was selected as performing well against the agreed set of quality indicators (referred to as hospital “A”), while the other was selected as average for the same set of indicators (referred to as hospital “B”). In all hospital A’s, two clinical micro systems were studied. In four countries maternity was one of the clinical micro systems under study^a^. The following micro systems were included: maternity, oncology, orthopaedics, elderly care, intensive care, and geriatrics. In all hospitals a specific quality improvement project was studied in detail over time, hence data from the micro level is included in all ten hospitals.

### Data collection

Data collection was conducted over a 12 month period (April 2011 to April 2012) by research teams in the respective countries according to the agreed study protocol [14] and agreed templates for a) semi-structured interview guides at meso and micro level, b) observation guide, and c) mapping of macro level socio-political context.

Data relating to the macro level in each country were mainly collected from documentary sources according to the agreed framework adapted and extended from those of other studies [23–25]. Topics covered in the macro level framework were: funding, access, regulation, accreditation, monitoring, information and resources available for quality work, and patient rights. In particular data relating to the regulation of quality were relevant in this study as they included whether or not quality of healthcare was defined nationally and/or regionally; how it was defined; whether CE, PS, PE was included; whether written policy documents specifying the quality of care existed; and what aspects of quality they addressed? Documentary sources such as legislation, regulation, and national strategies were used for this purpose. Data collection at the macro level did not include interviews or observation with national bodies for the purpose of understanding how quality was conceptualized. Written documents were considered suitable to review the ‘official’ quality definition communicated ‘downwards’ in the healthcare systems.

To guide the data collection and analysis at the meso and micro level we applied the six ‘challenges’ identified by the ‘Organizing for Quality’ study [26] (structure, culture, emotions, politics, education, and physical environment and technology) and added two challenges (leadership and external demands). These eight challenges formed the framework for the interview topic guides. All topics in the interview topic guide were linked to CE, PS, and PE when asking the informants about ‘quality’. To illustrate how this was performed at meso level we asked senior managers: *How does your hospital show that it values, rewards and celebrates ‘quality’? Do practitioners (doctors, nurses) and managers work well together to improve quality? How do you measure and report on quality within this hospital? (with regard to each of the dimensions CE, PS, PE).* An example of a question we asked at the micro level was: *How would you rate the hospital in terms of its overall quality [CE,PS,PE] and quality improvement compared to other similar hospitals? Is how you think about quality and quality improvement the same as that held by higher management in the hospital or other people working in this unit? [if not, how do these views differ?].*

A total of 383 interviews and 803 hours of observations (including 207 meetings relating to QI) were conducted at the meso and micro level (see Table 2). Informants included senior hospital managers, administrative staff, and health professionals with and without managerial responsibility. All interviews were audio-taped and transcribed in the native languages according to a standardized format. The research strategy of the QUASER-project is considered longitudinal as data collection included repeated observations and interviews conducted over a one year period, including re-interviews of the same informants a year after the first interview to validate findings and ask for changes and challenges during the last year. However, this paper does not reflect changes or development over time. Data collection at meso and micro level also included collection of documents such as hospital policy, annual reports, and review of hospital websites to collect data on how the hospitals described themselves with regards to PS, CE, PS [14]. Here we report on a subset of the larger study analyzing the findings in relations to the conceptualization of quality which reflects one out of seven main research questions in the QUASER project.

**Table 2:** **Summary of fieldwork undertaken (April 2011 to April 2012)**

Hospital	Meso-level			Tracer project			Micro-level		
	Ints.	Obs.	Mtgs.	Ints.	Obs.	Mtgs.	Ints.	Obs.	Mtgs.
The Netherlands a	37	90	25	9	65	19	9	130	26
The Netherlands b	36	100	31	15	31	7			
Sweden a	14	20	7	9	12	5	13	8	8
Sweden b	15	6	2	2	6	1			
England a	13	65	16	5	25	10	21	97	6
England b	24	20	7	5	10	3			
Portugal a	15	0	0	11	0	0	26	57	10
Portugal b	20	18	12	3	10	3			
Norway a*	18	2	3	7	2	1	25	20	2
Norway b**	25	2	1	6	7	2			
**TOTAL**	**217**	**323**	**104**	**72**	**168**	**51**	**94**	**312**	**52**

*in addition a focus group interview in the maternity micro system (3 participants) (Micro system Norway A), and a group interview (3 participants) (Tracer project Norway A).

**in addition 16 interviews, 12 hours observation, a focus group interview with 7 participants and 3 meetings at micro level in hospital B.

Key: Ints= the number of interviews conducted. Obs= the number of hours of practice observation. Mtgs= number of meetings observed.

If required, ethical approval was granted in each country and consent obtained from the involved informants. The following institutions approved the project: Norwegian Social Science Data Services, ref. 26636 (Norway); Regional Ethical Committee, Lindköping, ref. 2011/164-31 (Sweden); and NRES Committee South East Coast, Surrey, ref. 11/L010348 (England). Ethical permission for this study was not necessary under Dutch and Portuguese law as no patient data was collected. In the Portuguese hospitals, the board of directors authorized the collection of data, and the ethics committee was informed.

### Data analysis

**Figure 1:**
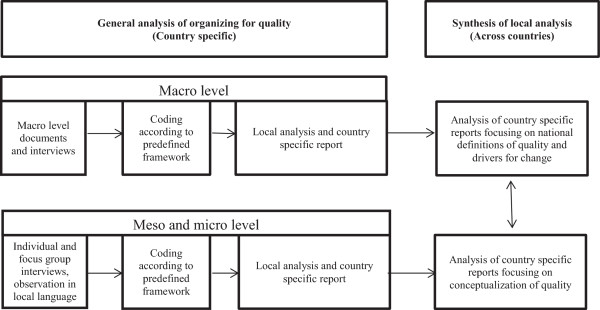
**Process from data collection to country specific analysis to cross country analysis.**

The data analysis consists of a two-step process: 1) the within country analysis of the total country specific data material, and 2) a cross country analysis synthesizing and comparing the five country specific analyses [27–29]. The first step is based on original findings in each country; the second step is based on qualitative meta-synthesis which is a qualitative study using the findings from other qualitative studies as data, linked by the same or a related topic (here conceptualization of quality) [28, 29]. The analytical process is presented in Figure 1, based on Hesselink et al. [30].

### Within country analysis

All transcribed interviews were uploaded in Nvivo or Atlas.ti and analysed by the country teams according to an agreed code-book based on predefined categories of the 8 challenges of our framework (structure, culture, emotions, politics, education, and physical environment and technology, leadership, and external demands). The analysis was developed into country specific reports and translated into English. In these reports all countries answered the overall research questions of the project for their respective country, based on the entire coded country specific data material. Agreement about the code-book was achieved before the analysis stage. Preliminary analysis and emerging themes were discussed in researcher meetings and QUASER consortium meetings with all partners face-to-face. All country specific reports included a sub-chapter on “*Conceptualization of quality*” where country specific data were analysed according to the three dimensions of quality – CE, PS, PE – related to the meso and micro level findings. For this part of the analysis the research team in each country analysed the meso level interviews with the senior managers in all hospitals to explore what quality dimensions senior managers talked about, what quality dimensions they considered to be most important, and whether other dimensions beyond CE, PS and PE emerged as important aspects of what they considered quality in healthcare. The same was carried out at the micro level, and in addition we analysed for differences between professional groups (managers, doctors, nurses). In addition all partners developed a country specific macro-level report to describe the socio-political context, including data on how the macro level defined quality.

### Cross country analysis

For the meta-synthesis, we used the five country specific macro level reports and the five country specific case study reports as data sources [28, 29]. We analysed the macro level reports in relation to how the quality was conceptualised at the macro level within the five countries, focusing on included dimensions; key policy documents where definitions are found; and additional quality dimensions emphasized. At the meso and micro level we analysed the country specific sections on “Conceptualization of quality” in the case study reports and we coded the data into categories of CE, PS, and PE. In addition, we analysed the case study reports to map additional material emerging from the country analysis illustrating how conceptualization of quality is shaped and communicated within and across hospitals (e.g. the role of: managers, professional communities, and other quality aspects such as ethical dilemmas and professional competence). In the meta-synthesis we also explored patterns across the six different micro systems, and compared differences and similarities between what senior managers, and other professional groups (doctors and nurses) include in their meaning of quality. Three researchers (SW, KAA, CvP) synthesised the data from the case reports and the macro level reports [28, 29, 31, 32]. The preliminary analysis was discussed in QUASER consortium meetings involving all partners, refinements made and then verified by researchers in each country.

## Results

The results are presented according to conceptualization of quality at a) the macro level, b) the meso level, and c) the micro level. Each section provides a table including quotes, categories and emerging themes to support the respective results.

### Conceptualization of quality at the macro (national policy) level

All five countries shared a conceptualization of quality at the macro level that incorporated the three quality dimensions of CE, PS, and PE (see Table 3). However, all macro level definitions and policy priorities included other dimensions of quality, in addition to CE, PS, and PE. Transparency on quality outcomes was emphasized in the Netherlands and Portugal; equity and access were emphasized in Norway and England; Portugal paid attention to accreditation and complaints; and Sweden emphasised equity and a systematic QI approach focusing on systems, organising, processes, and cooperation. England also included staff satisfaction and responding to emergencies as elements of the prioritized dimensions at the macro level.

**Table 3:** **Conceptualizations of quality at the macro level**

	England	Norway	Sweden	Netherland	Portugal
**Document** **source**	Health and Social Care Act, Department of Health	National Quality Strategy	National Board of Health’s Quality Regulation and The Health Care Act	The National Quality Act	National Health Plan
**Quality definition**	“*Over the last three years, the NHS has coalesced around a shared definition of quality. This was set out by Lord Darzi in the NHS Next Stage Review Leading Local Change as comprising three elements:*	“*The definition of quality is based on meeting the demands of society, meeting legislative requirements, and providing users with the best possible services from a professional perspective. For health and social services, high quality means that the services:*	*“The health and care services shall be conducted according to requirements for good care. This includes that it shall 1) be of good quality with high hygiene standard and consider patients’ need for safety in care and treatment; 2) be easy to access; 3) be based on respect for patient self-determination and integrity; 4) foster good contact between the patient and health and care professionals; 5) consider the patient need for continuity and safety in the healthcare services.”* (translated by authors) [33]:§2a.	*"The healthcare provider offers responsible care. Responsible care is defined as care of a good level, at least effective, efficient and patient-oriented care which is geared to the real needs of the patient."*	*“Quality in health (QeS) can be defined as the provision of affordable and equitable healthcare, with an excellent professional level, taking into account the available resources, while achieving the citizen’s adhesion and satisfaction (Saturno P et al., 1990). It also implies the adequacy of healthcare to the needs and expectations of citizens and the best possible performance.”*
	*•effectiveness of the treatment and care provided to patients*				
	*•the safety of treatment and care provided to patients*				
	*•the experience patients have of the treatment and care they receive.*” [34]:p.9, [20]:p.47.				
		*• Are effective*		(translated by authors) [35].	(translated by authors) [36]:p.2
		*• Are safe and secure*			
		*• Involve users and give them influence*			
		*• Are coordinated and continuous*			
		*• Utilize resources efficiently*			
		*• Are available and evenly distributed.”* [37]:p.12.			
**Additional quality dimensions and priorities**	Cleanliness, infection reduction, access, responding to emergencies, reduction of health inequalities, staff satisfaction.	Coordinated, integrated, appropriate resource use, available and equally distributed services.	Organizing and management, processes, equity, cooperation, systematic QI.	Timely, transparency on quality outcome, efficient.	Organizational quality, transparency, qualifications, accreditation, mobility, assessment, and complaints.
**Inclusion of CE, PS, PE**	Yes	Yes	Yes	Yes^a^	Yes

^a^The National Quality Act included CE and PE. Although PS is not explicitly mentioned in the regulation (National Quality Act), there is a strong emphasis on developing indicators on PS. The Minister of health defined quality as fast, good, safe and respectful in a letter to parliament [38]. The Quality act was made in 1996, when PS was not very high on the agenda; meanwhile it has become the main thing also in the Netherlands.

### Conceptualization of quality at the meso (hospital management) level

#### Clinical effectiveness, patient safety, patient experience

The background of the senior hospital managers varied in the study. Some were clinicians with backgrounds as nurses or doctors, while others had a managerial background. From our analysis we found only minor differences in the conceptualization of quality for senior hospital managers depending on their background. The results showed a broad perspective of quality articulated in policy documents and by the senior hospital managers, including the dimensions of CE, PS, CE, however efficiency and costs were also often included in the definition by managers.

The importance of the three quality dimensions varied to some degree across the hospitals, but they all matched the national definition. For example in Portugal and Norway, the main emphasis was on CE, while PS was the main dimension in the Netherlands since the national government required a patient safety system in each hospital. In the different hospitals, senior managers’ focus on CE related to level of professional competence, evidence-based practice, hospital size, and status as university hospital; while their attention to PS materialized in a large number of tools, such as safety ward rounds, prospective risk analysis, and blame-free safety incident reporting. The PE dimension was present in the public narrative (on websites, in strategic documents at hospital level, and in interviews), especially in England and the Netherlands, meaning that the hospitals presented themselves to the public and in the media as giving emphasis to all three quality dimensions. But in practice PE was not used as an important source in QI across all hospitals, and senior managers experienced PE as a difficult dimension to implement due to lack of relevant tools and knowledge. The latter was especially evident in the Norwegian hospitals.

#### External demands and quantifiable quality data

At the meso level the results showed a managerial approach emphasising control and measurement of quantifiable quality data, such as quality indicators and national targets. This was especially evident in England, where for example the board at England hospital A paid great interest in clinical quality measures after a restructuring process. The clinical measures were considered a more systematic approach to improve quality relying on accountability and assurance of results. The conceptualization of quality at the meso level in Norway, England, and the Netherlands was largely influenced by external factors, such as national targets, indicators, and campaigns. Norway has a growing attention to national quality indicators communicated from the macro level to the meso level. Here senior managers emphasized their own brokering role between macro and micro level, and how they shape attention to quality dimensions in their communication and leadership role, e.g. when they implemented a new national quality measure of perineal rupture rate in the maternity micro system. The external demands, in terms of achieving targets and measure quality, were not always seen as positive by the senior hospital managers and some experienced the measuring as the end and not as a mean to ensure quality. In the Netherlands many different external actors and stakeholders (such as insurance companies) pay extensive interest to quality and there was a large focus on transparency of quality outcomes. The hospitals in the Netherlands established numerous QI projects that often depended on fragmented external demands, and here quality was often thought of in terms of quality improvement projects.

#### Quality culture

At both Swedish hospitals the senior hospital managers conceptualized quality as a key task for doctors and nurses, and had implemented a model where all employees have specified time in their schedule for QI work, and mandatory inter-professional QI training, in addition to their ordinary clinical work. In three of the case hospitals (Sweden A, Portugal B, Norway A) we found a cultural approach to quality among senior hospital managers, emphasizing the importance of a continuous attention to establish a quality culture that integrated quality into professional clinical practice.

In Table 4 we illustrate how senior managers conceptualized quality and give examples of different dimensions included in their perspective.

**Table 4:** **Conceptualizations of quality at the meso level**

Data source	Quote	Category	Emerging themes
Senior manager Norway B	*‘Currently we talk about economy, yes, we still talk about that, but not economic aspects only. We talk a lot about professional development, patient quality, patient safety, how to improve patient pathways in an efficient manner, and how to solve the targets specified in the letter of assignment, such as waiting lists, priorities such as deadlines related to discharge summaries. So, when you ask me to compare with other hospitals, I think that all hospitals pay attention to patient safety, because we all know that if our patients and the next of kin are dissatisfied, we have to work against that. That is really tough’.*	CE, PS, PE	Economy, measures, professional development
Senior hospital manager Norway B	*‘There are too many ruptures. The Minister of Health wants to increase the attention to ruptures to decrease the rate. What we [managers] pay attention to is amplified. That’s how it is, we have to succeed’.*		External factor influence, the brokering role of managers in shaping attention to quality
The president of the board Portugal A	*‘A place of this size and its multidisciplinary nature should be on another level [treatment] and we should collaborate with clinical trials planned by other hospitals and countries’.*	CE	Hospital size and status - CE as being in the forefront of treatment and research
Senior manager England A	*‘So demand from the general public and also demand from organisations. Endless streams of targets to try and achieve, which again, they are there for quality, so we have measuring incident rates of thrombosis, pressure sores, all these sorts of things, which is good, and nutrition analysis on the ward, but sometimes these things are … almost the analysis is the means to an end and I think we’re trying to do these things to ensure quality, not just to ensure that we’ve met the targets, and there seems to be a focus on that’.*	CE, PS	Measuring quantifiable quality data, response to external demands
Senior manager England A	*‘With the recent organizational change, the board intends to replace luck with system, accountability, and assurance’.*		Quantifiable quality data
Ward manager Nether-land B	*‘I really need a project for it. Yes, now and again I work on it, just to give a subject item a bit of stimulus. But it’s not feasible to do it too often, in addition to all the other things we have to do’.*		Quality depend on fragmented external demands
Clinical manager Sweden A.	*‘I have been part of the journey that this clinic has done. We have done a long journey regarding process work and development of the clinic and we have come very far and it’s very stimulating to work, everyone is in that thought mode – improvement think’.*	CE, PS, PE	Quality culture

### Conceptualization of quality at the micro level

#### Between different clinical professions

In our exploration of variations between clinical professions, results from Portugal and Norway showed that the conceptualization of quality was strongly linked to professional roles and differed between professional groups, meaning that e.g. doctors, nurses, and midwives differed in what they articulated as important dimensions in their conceptualization of quality. Quality was always conceptualized as integral to professional work. Results from the English micro systems were similar, where the professionals conceptualised quality according to personal ideas and beliefs of what constituted high care quality. In comparison, Swedish nurses and doctors had a larger degree of shared conceptualization of quality. In all hospitals, with the exception of Sweden, we found that CE was the dominant quality dimension among the doctors who emphasised the evidence-based approach in their conceptualization of quality. Results from Norway, England, the Netherlands, and Portugal showed that nurses conceptualised quality with a strong link to PE in terms of caring and valuing patient-centred care, but less in terms of using PE for QI purposes. Nurses placed a premium on softer quality aspects, such as physical environment and having enough time to talk with the patients. Micro level staff were preoccupied with professional tasks, professional competence, evidence-based medicine, and caring for patients. The local adaptations, time pressure, facilities, cleaning, parking, and having time to provide what professionals personally believed to be high quality, emerged as key aspects of how professionals conceptualised quality beyond the CE, PS, and PE dimensions.

#### Between levels: meso-micro

In our study we explored differences in conceptualization between meso and micro level. In several countries, patient-centred care appeared as the quality “trade-off”. More specifically, this was related to priorities and cost reduction decided at the meso level. Micro level staff experienced that cost reductions and reorganizing of the services implied less room for patient-centred care, while tasks related to PS or CE were not considered “traded off” in the same way when the financial situation was difficult. The experiences of trading off the patient-centred care, (e.g. reduced time for care, limited options for involving relatives, less time for guiding mothers in breast feeding) caused frustration and ethical dilemmas for the professionals at the micro level. Professionals at micro level expressed a perception of a different conceptualization of quality between themselves and the meso level managers, who often were accused of thinking in economic terms and measurement of quantifiable quality variables.

#### Between institutional contexts

Nurses and doctors were strongly influenced by their professional culture and pride, organisational identity, and their respective professional communities in shaping their conceptualization of quality. The results showed significant differences related to hospital context. In the large university hospitals, such as Portugal A and Norway B, the conceptualization among micro level staff was built on strong organisational identities and professional pride being at the forefront of clinical research. The evidence found in national and international professional communities was often more trusted than evidence presented by national governmental bodies. The continuous search for new evidence based knowledge on improved diagnostic methods, tools, technologies, and the ability to handle complexity (treatment) was closely related to a conceptualization of quality as CE - not PS or PE. This implied a search for improved care quality by improving CE (of already highly specialized services), with less emphasis on other quality dimensions. In some of the smaller hospitals, more attention was directed towards building an organizational culture with an identity of shared values (Sweden A and Norway A). In these hospitals the diversity in conceptualization between professional groups was less visible, as were conflicts and professional competition.

#### Between clinical services

The study included six different types of clinical micro systems and the results showed that the within-professions quality conceptualization differed according to services provided (maternity, oncology, orthopaedics, elderly care, intensive care, geriatrics). To exemplify, in oncology and palliative care, the key elements of quality mentioned by nurses and doctors were related to ethical challenges (providing strong medication to palliative patients with risk of over dose versus risk of severe pain) and the time component. While in other micro systems such as maternity or intensive care the nurses and doctors were more preoccupied with rapid decision making, competence, and complying with professional procedures as a basis for providing high quality care. Table 5 illustrates how professionals at the micro level conceptualized quality.

**Table 5:** **Conceptualizations of quality at the micro level**

Data source	Quote	Category	Emerging themes
Nurse, Netherland B	*‘We can talk about quality of course, but first of all they must see to it that people don’t have to wait months to be admitted, that the patient understands what we’re going to do, and that all the necessary things are available. That’s what I call quality. Basic things that are often not properly sorted out here and that exasperates me day in, day out’.*	PE	
Doctor Portugal A	*‘In terms of clinical effectiveness, initially all patients had to be cured, but as this is impossible in terms of pathology and results, we want the best effectiveness outcome appropriate for the situation’.*	CE	
Midwife Sweden A	*‘I think that we more or less work as a team, we are posing question and counter question to each other and it’s not only the doctor that is deciding if we should do this or that, even though we should respect each other’s knowledge and it’s as for the doctor that he or she follows the clinical guideline, as it is for us to follow it’.*		Shared conceptuali-zation of quality between professional groups
Department midwife Norway A	*‘It is obvious that their [managers] mind-set is more related to money, while we think more directly about the patients on a daily basis’.*		Different quality perspective between managers, healthcare professionals
Midwife Norway B	*‘The managers remind us about coding all the time. “Remember to code [codes in IT system related to financial reimbursement], remember to code, remember to code”. That is how we are funded. At almost all our department meetings, an expert is present to show us how to do it and how we can improve our coding. “We lose a lot of money if you don’t do this or if you do that”. They tell us all the time. I am a midwife, I am not an economist. I am not “running a shop”’.*		Different quality perspective between managers, healthcare professionals
Member of the infection control committee Portugal A	*‘Aspects related to the human being as a whole, in general, they are not handled. Human beings are a whole and not just the pneumonia or broken leg that took them to the hospital…it is this idea of the whole that fails in this society we live in and this is also reflected in treatment, which is very technological. We have good equipment, excellent endoscopes, excellent neurosurgeons, we have excellent surgeons, but dealing with that situation. However, the patient is a whole and it’s that part that I think quality needs to worry about, which I think is one of its failings’.*	CE	Highly specialized services caused lack of holistic perspective on quality beyond CE
Chief medical doctor palliative team Norway A	*‘Time is our luxury, we have enough time, and if we don’t have time we are useless…. The real indicator of our success is the patients’ subjective experience’.*	PE	Quality dimensions vary between clinical services

To summarize, the micro level results showed a striking diversity of conceptualizations of quality. There were different conceptualizations of quality across system levels, types of services, and professional groups.

## Discussion

All participating countries had a macro level definition of quality incorporating CE, PS, and PE. Additional dimensions were included depending on the priorities within the country such as access, equity, and transparency. Macro level attention was strongly related to quality measurement, which is in line with other studies of how the national bodies approach quality [13]. The quality dimensions and approaches were communicated via policy documents, national strategies, regulation, targets, quality indicators, and campaigns, and these influenced how quality was conceptualized and enacted, especially among the senior hospital managers. Our study demonstrated a substantial influence from the macro level demands on senior hospital managers’ approach to quality. Senior hospital managers seemed to adopt the national definitions and related their quality approach to quality indicators, targets, and campaigns; these incentives were important drivers of quality conceptualization (including CE, PS, PE); and promoted QI activities in the different hospital contexts.

Similar to other studies [5–7], the micro level conceptualization of quality in this study differed from both the meso and macro level definitions and conceptualizations, particularly for nurses where the quality language was about patient-centered care and time pressures, facilities, car parking for patients and the ability to provide what they believed to be high quality care. Our results support a recent Swedish study demonstrating that nurses’ perception of high quality care was strongly related to work environment factors of having enough resources and staffing, and competent leadership [39]. The doctors’ view on the other hand with a clear link to CE was associated with professional competence [5], skills and ability to provide complex treatment. Doctors’ trust in evidence based medicine [40] appeared as a pillar for their perception of delivering high quality services. There is a lack of findings related to collaboration, team work, and inter-professional training [41, 42] (except from Sweden) at the micro level which indicate that quality is mainly understood and shared within professional paradigms, services, or within system level, which is challenging in highly specialized services depending on hand-overs, information transfer, and coordination [43, 44] to provide and improve care quality.

Literature on improving care quality often emphasizes the role of senior hospital managers in terms of facilitating QI [45–48]. Our study confirms this picture by documenting how senior hospital managers influence QI work, based on how they articulate quality dimensions. Managers articulated a broad quality conceptualization including CE, PS and PE, but also included efficiency and costs. There seemed to be a need for budget balance before quality entered the managerial agenda and this influenced micro level staff perceptions of their managers’ attitude to QI which was seen as narrowly focusing on quality measurement and financial incentives. The managers’ attention on the control and measurement of quantifiable quality variables, accountability, and assurance can be interpreted as a response to external macro level demands. But it can also be interpreted as a potential source of conflict, as our results show a gap between what constitutes the meaning of quality at the meso and micro level [49].

The study highlights the important influence from professional culture and communities in socialising doctors and nurses [40, 50] in common conceptualizations of quality. Our findings echo those of other studies illustrating the diversity between professional groups and managers [1, 5, 8], but furthermore links the professional shaping of quality conceptualization to input from the national and international professional communities, which was often considered more trustworthy than input from national healthcare bodies. The variety of nursing, medical, and managerial conceptualization of quality may impede managers’ QI strategies and practices [1, 49].

### Limitations

The conceptualization of ‘quality’ used in the QUASER project (clinical effectiveness, patient safety and patient experience) [14] emerged as a limitation in our analysis. The study demonstrated how other important dimensions emerged in our data (for example, efficiency, time, economic issues). A broader conceptualization would have enabled a more thorough and consistent exploration of these dimensions.

Patients have a view of quality which often differs from professionals’ conceptualization of quality [51–53]. Patients’ conceptualizations were not included in this study [14, 54]. To achieve a deeper understanding of what patients include and value in their perception of quality in healthcare, future studies should include the patient perspectives on quality conceptualization [12].

Our study has not analyzed in depth the association between senior managers’ background in relation to their conceptualization of quality. Our results showed a relatively homogenous response from the senior hospital managers, however we believe further research is needed to provide a deeper understanding of how clinical, non-clinical, managerial background and work experience from other industries or sectors influence how senior managers conceptualize and approach quality in health care.

## Conclusion

This multi-level case study of hospitals across Europe does not provide a representative picture of generalizable quality conceptualizations across all European countries, hospital, and professional groups. However, our qualitative approach enabled an investigation of diversity in quality conceptualizations across different settings and groups which is of relevance for understanding QI processes, implementing improvement efforts, and bridging language and meaning about quality between system levels, professions, and clinical services [1, 5, 49, 55].

We conclude that quality is a contested concept where conceptualization varies significantly between publicly-celebrated organisational policy and images, and the ability to translate it into operational practice. The study showed a managerial alignment challenge as the conceptualization differed across system levels (macro-meso-micro), among professional groups (nurses, doctors, managers), and between the studied micro systems (maternity, oncology, orthopaedics, elderly care, intensive care, and geriatrics). The macro level conceptualizations included CE, PS, and PE as equal quality dimensions. CE was the most important dimension in the conceptualization of quality at meso and micro level, followed by the PS dimension. To a varying degree, PE was on the agenda, but overall our study supports Doyle et al.’s [56] conclusion of a need to adopt PE as a central pillar of quality in healthcare in order to take advantage of patient involvement and experiences as a source in QI.

### Implications

Based on our analysis and interpretations our study revealed two key lessons for senior hospital managers.

First, important macro level institutions in all countries defined quality, and the conceptualization at the meso level involved a broad perspective (CE, PS, PE) often in line with macro level quality definitions, policy or targets. However, the conceptualizations at the micro level varied and largely depended on professional group, type of service, and on softer quality dimensions beyond CE, PS, and PE. The ability of senior hospital managers to translate macro level demands into comprehensible information, empower staff, and prevent micro level compromises related to professionals’ conceptualization of quality care, is key for senior hospital manager in fostering collaboration and coordinated action in QI.

Secondly, the differing conceptualizations of quality across levels and between professions within the hospitals, suggests a need to establish arenas to prevent a professional silo approach to QI, and to take advantage of the strong commitment and trust healthcare professionals have to their professional and scientific communities. Different actors have different roles in QI, and senior hospital managers’ QI strategies should not be a matter of choosing the “right” perspective or definition of quality. Managers need to acknowledge the variety in quality concepts and need to build a minimum shared understanding (including understanding of how other professionals understand quality [57]) in facilitating QI processes. In practice this could be e.g. establishing inter-professional teams in quality improvement projects as a way to build better understanding between professional perspectives and to take advantage of their different background and competence to improve care quality. On the other hand, hospital managers also need to acknowledge the fact that a predominance of some quality views over others (e.g. clinical effectiveness) could be counterproductive for improving quality of care, and that a too broad quality conceptualization could end up as superficial and lack in-depth knowledge.

## Endnote

^a^As maternity care in the Netherlands is organized differently than the other countries (mostly home delivery under supervision of midwifes), a more comparable case study on the Oncological department was chosen. In Norway, a maternity micro system was included also in hospital B due to a request from the hospital itself.

## Abbreviations

CE: Clinical effectiveness
PS: Patient safety
PE: Patient experience
QI: Quality improvement.

## References

[1] Braithwaite J, Westbrook MT, Robinson M, Michael S, Pirone C, Robinson P (2011). Improving patient safety: the comparative views of patient safety specialists, workforce staff and managers. BMJ Qual Safe.

[2] Greenfield D, Nugus P, Travaglia J, Braithwaite J (2011). Factors that shape the development of interprofessional improvement initiatives in health organizations. BMJ Qual Safe.

[3] Institute of Medicine: To Err is Human (2000). Building a Safer Health System.

[4] Institute of Medicine (2001). Crossing the Quality Chasm.

[5] Travaglia JF, Nugus PI, Greenfield D, Westbrook JI, Braitwaite J (2012). Visualising differences in professionals’ perspectives on quality and safety. BMJ Qual Safe.

[6] Rosenfeld K, Wenger NS (2000). Measuring quality in end-of-life care. Clin Geriatr Med.

[7] Attree M (1993). An analysis of the concept “quality” as it relates to contemporary nursing care. Int J Nurs Stud.

[8] Travaglia JF, Westbrook MT, Braithwaite J (2009). Implementation of a patient safety incident management system as viewed by doctors, nurses and allied health professionals. Health (London).

[9] Attree M (1996). Towards a conceptual model “Quality Care”. Int J Nurs Stud.

[10] Atree M (2005). Nursing agency and governance: registered nurses’ perceptions. J Nurs Manag.

[11] Attree M (2008). Patients’ and relatives’ experiences of “Good” and “not so good” quality care. J Adv Nurs.

[12] Levine R, Shore K, Lubalin J, Garfinkel S, Hurtado M, Carman K (2012). Comparing physician and patient perception of quality in ambulatory care. International J Qual Health Care.

[13] Mearns A, Vesseur J, Hamblin R, Long P, Den Ouden L (2011). Classifying indicators of quality: a collaboration between Dutch and English regulators. International J Qual Health Care.

[14] Robert GB, Anderson JE, Burnett SJ, Aase K, Andersson-Gare B, Bal R, Calltorp J, Nunes F, Weggelaar AM, Vincent CA, Fulop NJ, QUASER team (2011). A longitudinal, multi-level comparative study of quality and safety in European hospitals: the QUASER study protocol. BMC Health Serv Res.

[15] *The QUASER GUIDE*. [https://www.ucl.ac.uk/dahr/quaser]

[16] House R, Rousseau DM, Thomas-Hunt M (1995). The meso-paradigm: a framework for the integration of micro and macro organization. Res Organizational Behav.

[17] AHRQ[https://info.ahrq.gov/]

[18] Vincent C (2006). Patient Safety.

[19] Coulter A (2005). What do patients and the public want from primary care?. Br Med J.

[20] Department of Health (2008). High Quality Care For All NHS Next Stage Review Final Report.

[21] European Commission C (2008)4598 of August 2008 (2009). Work Programme 2009. Cooperation Theme 1 Health.

[22] Burnett S, Renz A, Wiig S, Fernandes A, Weggelaar AM, Calltorp J, Anderson JE, Robert G, Vincent C, Fulop N (2013). Prospects for comparing European hospitals in terms of quality and safety: lessons from a comparative study in five countries. Int J Qual Health Care.

[23] Wendt C (2009). Mapping European healthcare systems: A comparative analysis of financing, service provision and access to healthcare. J Eur Social Policy.

[24] Grosse-Tebble S, Figueras J (2005). WHO European Observatory on Health Systems and Policies. Snapshots of Health Systems.

[25] Commonwealth Fund (2010). International Profiles of Health Care Systems.

[26] Bate P, Mendel P, Robert G (2008). Organizing for Quality.

[27] Yin RK (2003). Case Study Research. Design and Methods.

[28] Zimmer L (2006). Qualitative meta-synthesis: a question of dialoguing with texts. J Adv Nurs.

[29] Finfgeld-Connett D (2010). Generalizability and transferability of meta-synthesis research findings. J Adv Nurs.

[30] Hesselink G, Flink M, Olsson M, Barach P, Dudzik-Urbaniak E, Orrego C, Toccafondi G, Kalkman C, Johnson JK, Schoonhoven L, Vernooij-Dassen M, Wollersheim H, on behalf of the European HANDOVER Research Collaborative (2012). Are patients discharged with care? A qualitative study of perceptions and experiences of patients, family members and care provider. BMJ Qual Safe.

[31] Walsh D, Downe S (2005). Meta-synthesis method for qualitative research: a literature review. J Adv Nurs.

[32] Sandelowski M, Barroso J (2007). Handbook for Synthesizing Qualitative Research.

[33] Health and Care act (1982) (Sweden): *Helso-och sjukvårdslag (1982:763)*. https://lagen.nu/1982:763

[34] National Quality Board (2011). Quality Governance in the NHS. National Quality Board ‒A Guide for Provider Boards.

[35] National Quality Act (1996) (the Netherlands)http://wetten.overheid.nl/BWBR0007850/geldigheidsdatum_22-01-2013

[36] Direção Geral de Saúde (Portugal) (2012). National Health Plan 2012–2016.

[37] Directorate of Health (2005). National Strategy for Quality Improvement in Health and Social Services (2005–2015).

[38] Schippers EI (2013). Ministry of Health, Welfare and Sport, Letter to the Parliament About Patient Safety.

[39] Alenius LS, Tishelman C, Runesdotter S, Lindquist R (2013). Staffing and resource adequacy strongly related to RN’s assessment of patient safety: a national study of RNs working in acute-care hospitals in Sweden. BMJ Qual Safe.

[40] Mascia D, Cicchetti A, Damiani G (2013). “Us and Them”: a social network analysis of physicians’ professional networks and their attitudes towards EBM. BMC Health Serv Res.

[41] Salas E, Rosen MA (2013). Building high reliability teams: progress and some reflections on team training. BMJ Qual Safe.

[42] Brock D, Abu-Rish E, Chiu CR (2013). Interprofessional education in team communication: working together to improve patient safety. BMJ Qual Safe.

[43] Cohen MD, Hilligoss PB (2010). The published literature on handoffs in hospitals: deficiencies identified in an extensive review. Qual Safe Health Care.

[44] Drachsler H, Kicken W, van der Klink M, Stoyanov S, Boshuizen HPA, Barach P (2012). The Handover Toolbox: a knowledge exchange and training platform for improving patient care. BMJ Qual Safe.

[45] Leape L, Berwick D, Clancy C, Conway J, Gluck P, Guest J, Lawrence D, Morath J, O’Leary D, O’Neill P, Pinakiewicz D, Isaac T (2009). Transforming healthcare: a safety imperative. Qual Safe Health Care.

[46] Levey S, Vaughn T, Koepke M, Moore D, Lehrman W, Sinha S (2007). Hospital leadership and quality improvement: Rhetoric versus reality. Patient Safe.

[47] Kunzle B, Kolbe M, Grote G (2010). Ensuring patient safety through effective leadership behavior: A literature review. Safe Sci.

[48] Øvretveit J (2010). Improvement leaders: what do they and should they do? A summary of a review of research. Qual Safety in Health Care.

[49] Okhuysen GA, Bechky B (2009). Coordination in organizations: An integrative perspective. Acad Manag Annals.

[50] Gherardi S, Nicolini D (2000). The organizational learning of safety in communities of practice. J Manag Inquiry.

[51] Aanjesen KA, Skudal KE, Iversen HH, Bjertnæs ØA, Kjøllesdal JG, Lindahl AK (2012). Kunnskapssenteret. PasOpp-rapport nr 7–2012.

[52] Ocloo JE (2010). Harmed patients gaining voice: Challenging dominant perspectives in the construction of medical harm and patient safety reform. Soc Sci Med.

[53] King A, Daniels J, Lim J, Cochrane DD, Taylor A, Ansermino JM (2010). Time to listen: a review of methods to solicit patient reports of adverse events. Qual Saf Health Care.

[54] Wiig S, Aase K, Storm M, Gjestsen M, Harthug S, Solheim M, Robert G, Fulop N (2013). Investigating the use of patient involvement and patient experiences in quality improvement in Norway: reality or rhetoric?. BMC Health Serv Res.

[55] Faraj S, Xiao Y (2006). Coordination in fast-response organizations. Manag Sci.

[56] Doyle C, Lennox L, Bell D (2013). A systematic review of evidence on the links between patient experience and clinical safety and effectiveness. BMJ Open.

[57] Huber G, Lewis K (2010). Cross-understanding: Implications for group cognition and performance. Acad Manage Rev.

[CR58] The pre-publication history for this paper can be accessed here:http://www.biomedcentral.com/1472-6963/14/478/prepub

